# Acute Respiratory and Renal Failure Following Delayed Recognition of T-cell Lymphoblastic Leukemia in a Previously Healthy Child

**DOI:** 10.7759/cureus.108305

**Published:** 2026-05-05

**Authors:** Olivia R Kaufman, Christopher M Ahmad, Samira Haberman, Ethan Speer, Curtis King

**Affiliations:** 1 Medicine, Kansas City University of Medicine and Biosciences, Joplin, USA; 2 Primary Care, Kansas City University of Medicine and Biosciences, Joplin, USA

**Keywords:** acute kidney injury, acute respiratory failure, chemotherapy complication, continuous renal replacement therapy, hyperleukocytosis, multidisciplinary management, pediatric mediastinal mass, pediatric oncology critical care, t-cell lymphoblastic leukemia, tumor lysis syndrome

## Abstract

T-cell acute lymphoblastic leukemia (T-ALL) is an aggressive pediatric hematologic malignancy that frequently presents with mediastinal involvement. Delayed recognition can result in rapid clinical deterioration due to airway compression, metabolic derangements, and treatment-related complications. We report the case of a previously healthy 12-year-old male who developed acute hypoxemic respiratory failure secondary to a large mediastinal mass initially treated as pneumonia, in whom imaging demonstrated a large anterior mediastinal mass with airway compression and a malignant right-sided pleural effusion with the diagnosis of T-ALL. The hospital course was complicated by severe tumor lysis syndrome resulting in oligo-anuric acute kidney injury, recurrent respiratory failure with acute lung injury, chemotherapy-induced pancytopenia, neutropenic enterocolitis, venous thrombosis, and persistent *Candida albicans* fungemia. Despite aggressive multidisciplinary management, the patient developed progressive multiorgan failure, and on the 46th day of hospitalization, he died from refractory fungal sepsis. This case underscores the importance of early chest imaging in pediatric patients with persistent respiratory symptoms unresponsive to antibiotics. It highlights the complexity of managing overlapping oncologic and critical care emergencies in children with T-ALL.

## Introduction

T-cell acute lymphoblastic leukemia (T-ALL) is an aggressive hematologic malignancy that accounts for approximately 12-15% of pediatric acute lymphoblastic leukemia cases and is frequently associated with bulky anterior mediastinal disease [1]. Mediastinal involvement may lead to airway compression, pleural and pericardial effusions, and cardiopulmonary compromise, placing affected patients at risk for rapid clinical deterioration [2].

Early symptoms of T-ALL, including cough, dyspnea, fatigue, or decreased exercise tolerance, are often nonspecific and may resemble common respiratory infections [3]. In children who otherwise appear healthy, these symptoms may initially be attributed to pneumonia or a viral illness, delaying appropriate diagnostic imaging and hematologic evaluation [4]. Failure to recognize an underlying mediastinal process can result in sudden airway compromise and severe metabolic abnormalities related to markedly elevated white blood cell counts (hyperleukocytosis) or tumor lysis syndrome (TLS) and the need for emergent intensive care interventions [5].

Patients with anterior mediastinal masses are particularly vulnerable during sedation and airway manipulation. Supine positioning, sedation, and loss of muscle tone can worsen dynamic airway compression and impair venous return due to increased extrinsic pressure on the tracheobronchial tree and great vessels. These physiologic changes may precipitate rapid respiratory or cardiovascular decompensation, particularly in patients with significant tumor burden.

TLS represents a life-threatening oncologic emergency characterized by rapid cellular breakdown leading to electrolyte abnormalities, acute kidney injury, and multiorgan dysfunction [6]. It is characterized by hyperuricemia, hyperkalemia, hyperphosphatemia, and secondary hypocalcemia, leading to cardiac arrhythmias, seizures, and even death [7]. Patients with T-ALL and large mediastinal masses are at particularly high risk due to substantial tumor burden and steroid sensitivity [7,8]. In severe cases, renal replacement therapy may be required, further complicating the clinical course [9].

We present the case of a previously healthy child with undiagnosed T-ALL whose initial respiratory symptoms were treated as pneumonia, resulting in delayed recognition of a large mediastinal mass. The patient developed acute respiratory failure, severe TLS, acute kidney injury, and subsequent multisystem complications during induction therapy, ultimately culminating in fatal fungal sepsis. This case highlights the importance of early imaging in pediatric patients with persistent respiratory symptoms and underscores the need for rapid, multidisciplinary coordination when managing pediatric oncologic emergencies.

## Case presentation

A previously healthy 12-year-old male presented with several months of progressive fatigue, decreased exercise tolerance, and unintentional weight loss. Two weeks prior to admission, he was treated with azithromycin for presumed community-acquired pneumonia without clinical improvement. In retrospect, the persistence of symptoms despite antibiotic therapy, combined with progressive fatigue and weight loss, represented clinical features that warranted earlier imaging to evaluate for an alternative underlying etiology. On the day of presentation, while attending a soccer camp, he developed acute cyanosis and became minimally responsive. He was transported by private vehicle to the emergency department, where his oxygen saturation was approximately 70%.

On arrival, the patient exhibited severe respiratory distress and altered mental status, necessitating emergent endotracheal intubation for acute hypoxemic respiratory failure. Arterial blood gas analysis demonstrated severe metabolic acidosis with a pH of 7.03 and a base deficit of −19. Initial laboratory evaluation revealed profound hyperleukocytosis (white blood cell count 169,000/µL), anemia (hemoglobin 9.4 g/dL), thrombocytopenia (platelets 82,000/µL), and hyperglycemia (296 mg/dL), raising concern for an underlying hematologic malignancy.

**Figure 1:**
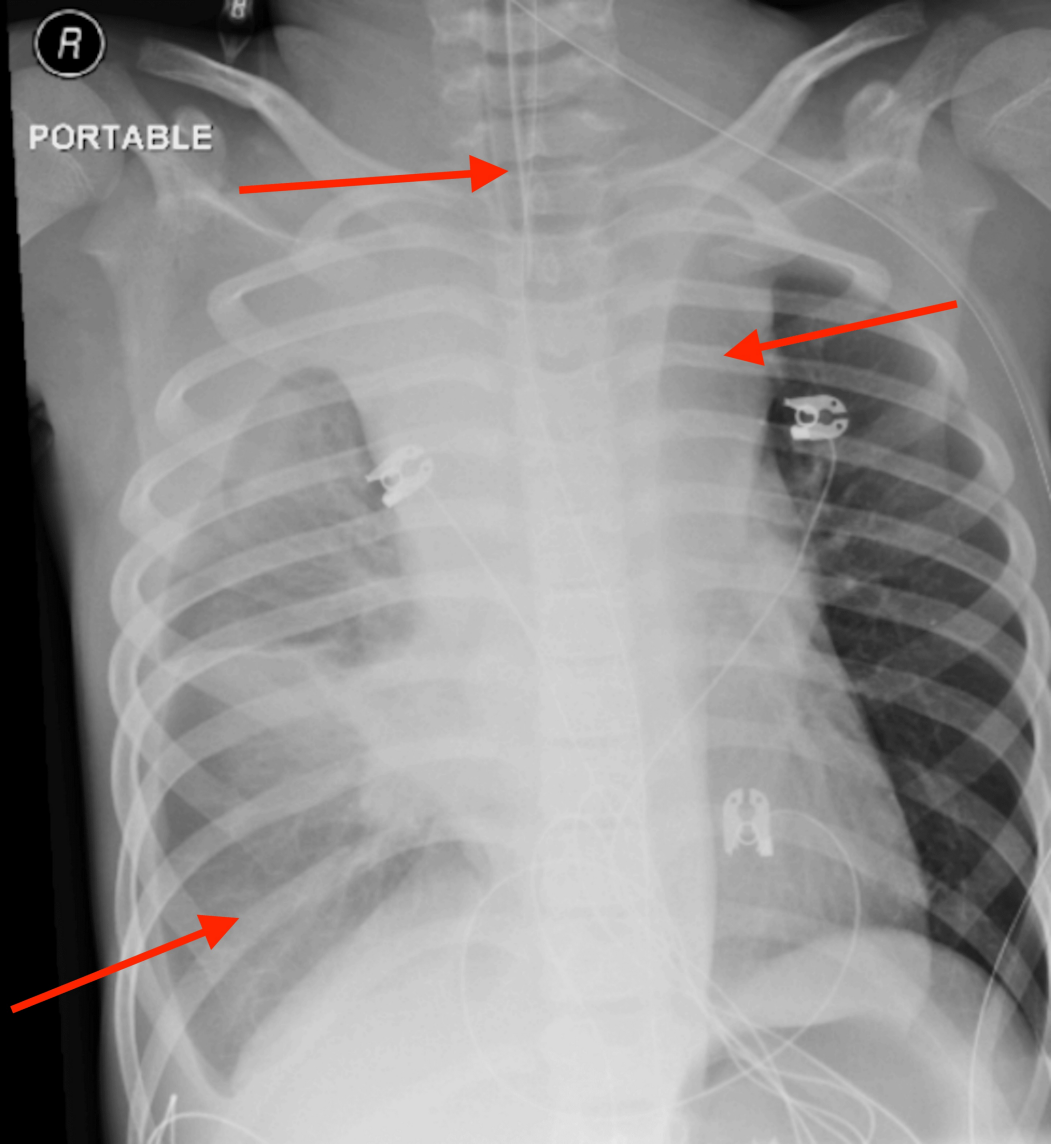
Portable supine anteroposterior chest radiograph The image demonstrates a widened mediastinal silhouette, right-sided pleural effusion, and associated right lung atelectasis (red arrows). These findings are consistent with a space-occupying mediastinal process with suspected mass effect on the central airway.

Chest imaging demonstrated marked mediastinal widening and a large right-sided pleural effusion. The patient was transferred to a tertiary pediatric intensive care unit for further management. Subsequent imaging confirmed a large anterior mediastinal mass causing tracheal compression with associated malignant pleural effusion (Figure 1). A right-sided chest tube was placed on hospital day (HD) 2 for pleural fluid drainage. Given the known risk of critical airway compromise and potential airway collapse associated with anterior mediastinal masses, airway management and sedation were approached cautiously in coordination with pediatric critical care, oncology, and anesthesiology teams [10].

Bone marrow biopsy revealed T-ALL with approximately 88% blasts. Cytogenetic analysis demonstrated t(7;10)(q34;q24), and cerebrospinal fluid analysis was negative for central nervous system involvement. The patient was initiated on induction chemotherapy per the Children’s Oncology Group protocol AALL0434 following stabilization, with early corticosteroid administration resulting in partial radiographic improvement of the mediastinal mass.

Shortly after initiation of therapy, the patient developed severe TLS characterized by hyperphosphatemia, hypocalcemia, rising uric acid levels, and oligo-anuric acute kidney injury. Despite aggressive medical management with intravenous hydration, allopurinol, and multiple doses of rasburicase, renal function continued to deteriorate (Table 1). Continuous renal replacement therapy was initiated, followed by transition to intermittent acute hemodialysis via a right femoral venous catheter due to initial improvement.

**Table 1:** Renal and metabolic laboratory trends across hospital course Dashes indicate laboratory values not available in the medical record for that date/time. Reference ranges are approximate standard pediatric/adolescent clinical ranges and may vary slightly by institution. BUN: blood urea nitrogen, LDH: lactate dehydrogenase

Laboratory tests	Reference range	Day 1	Day 5	Day 14	Day 18	Day 29	Day 32	Day 33 (AM)	Day 33 (PM)	Day 34 (AM)	Day 34 (PM)	Day 35 (AM)	Day 35 (PM)	Day 36
K⁺ (mEq/L)	3.5–5.0	4.0	—	—	3.3	3.1	5.0	4.5	3.5	3.7	3.5	3.6	3.1	3.5
Creatinine (mg/dL)	0.5–1.0	0.7	2.2	2.2	3.8	2.4	—	2.3	1.2	1.5	1.1	1.4	1.0	1.2
BUN (mg/dL)	7–20	14	49	45	83	96	111	88	38	53	40	58	39	51
Phosphorus (mg/dL)	3.0–5.5	5.8	—	—	4.7	—	—	6.3	—	4.0	—	3.5	—	3.0
Calcium (mg/dL)	8.5–10.5	7.5	—	—	7.9	—	—	—	8.0	—	—	—	—	—
Uric Acid (mg/dL)	3.0–7.0	6.9	—	—	—	—	—	—	—	—	—	—	—	—
LDH (U/L)	140–280	1304	—	—	—	—	—	—	—	—	—	—	—	—
Glucose (mg/dL)	70-140	296	—	—	78	115	105	122	113	113	119	118	110	161

**Figure 2:**
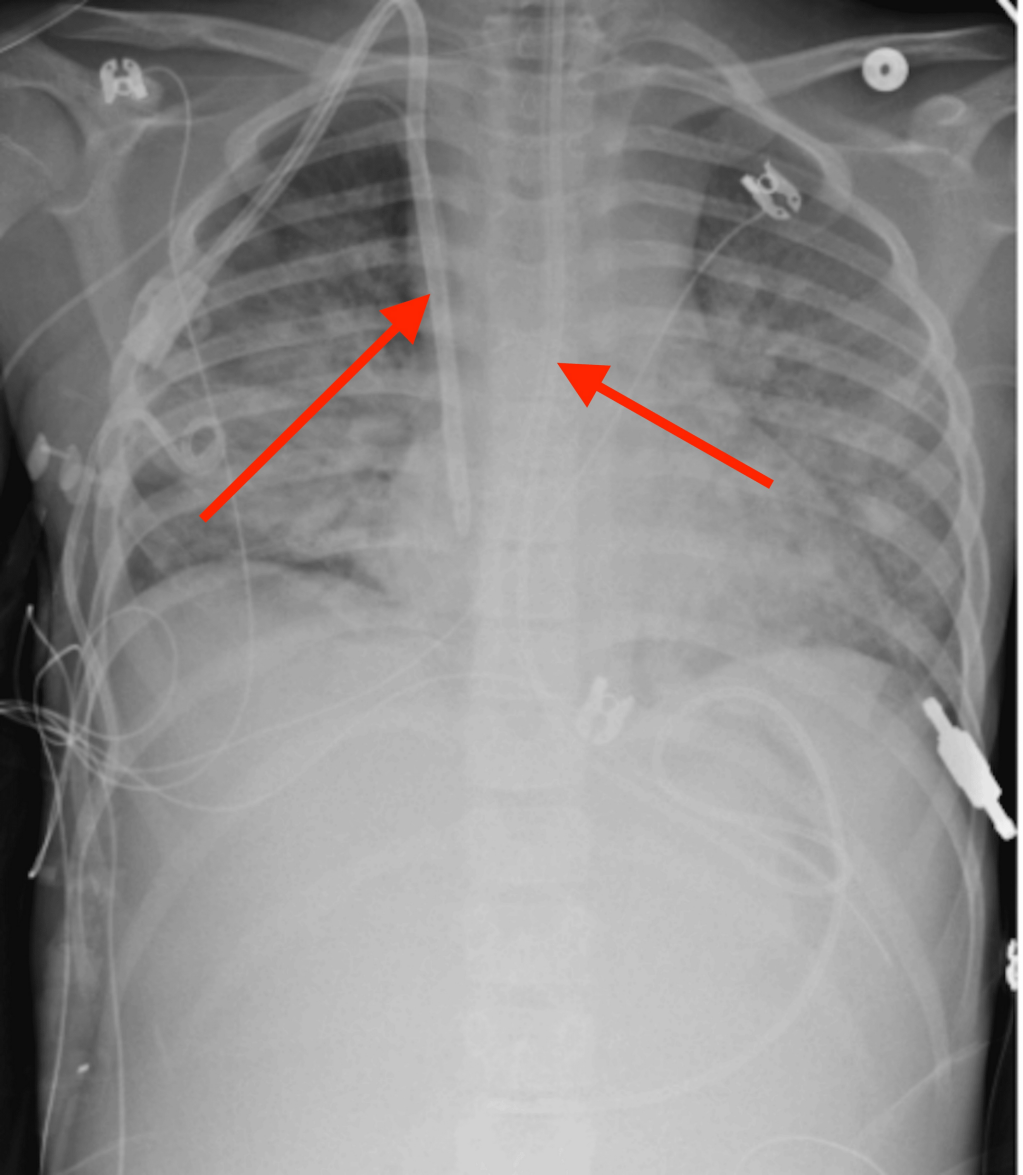
Portable supine anteroposterior chest radiograph This image demonstrates diffuse bilateral patchy airspace opacities with support devices in place, including an endotracheal tube and central venous catheter (red arrows).

The hospital course was further complicated by recurrent respiratory deterioration following initial extubation on HD 5 (Figure 2).

**Figure 3:**
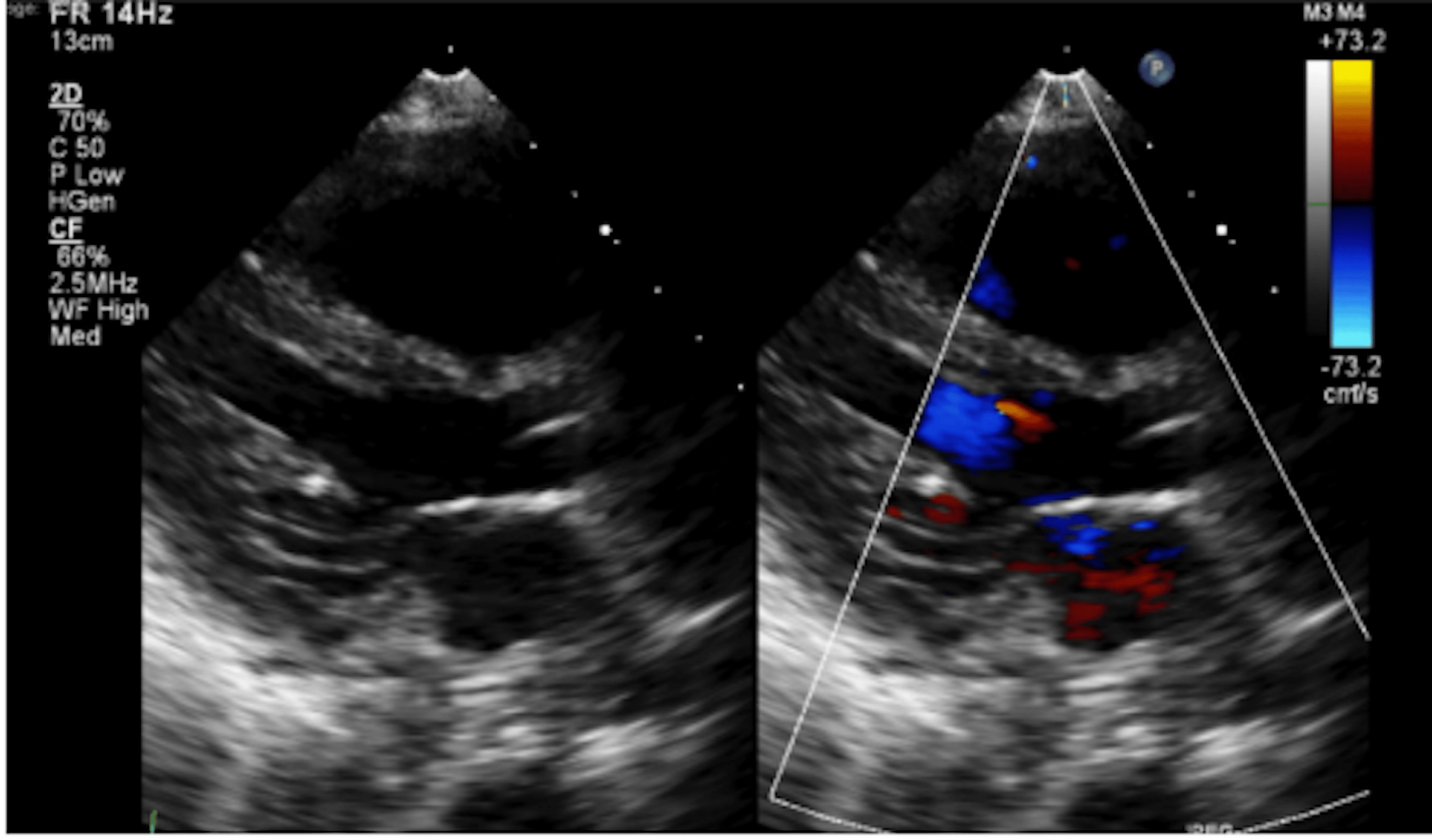
Bedside transthoracic echocardiography with color Doppler This image, performed during advanced respiratory failure, demonstrates cardiac function and flow patterns, providing a supportive hemodynamic assessment in the setting of multiorgan dysfunction and critical illness.

The patient developed acute lung injury and features consistent with acute respiratory distress syndrome in the setting of capillary leak, fluid overload, and systemic inflammation, requiring escalation of respiratory support, including bilevel positive airway pressure during dialysis sessions (Figure 3).

Additional complications included chemotherapy-induced pancytopenia (Table 2), neutropenic enterocolitis with radiographic evidence of bowel wall pneumatosis, and a right axillary vein thrombosis treated with therapeutic anticoagulation.

**Table 2:** Key laboratory hematologic trends across hospital course Dashes indicate laboratory values not available in the medical record for that date/time. Reference ranges are approximate standard pediatric/adolescent clinical ranges and may vary slightly by institution.

Laboratory test	Reference range	Day 1 (presentation)	Day 5	Day 14 (cytopenic nadir)	Day 18	Day 29	Day 32 (peak AKI)	Day 33 (AM)	Day 33 (PM)	Day 34 (AM)	Day 34 (PM)	Day 35 (AM)	Day 35 (PM)	Day 36
WBC (K/µL)	4.5–13.5	169	1.0	0.0	0.0	—	—	12.3	—	2.5	—	0.3	—	0.1
Hemoglobin (g/dL)	12–14	9.4	—	—	8.2	8.0	—	8.3	—	7.8	15.4	9.2	—	7.3
Platelets (K/µL)	150–400	82	51	21	14	—	93	62	43	61	17	51	23	33

Infectious complications emerged during the neutropenic phase of induction therapy. At presentation, the patient was started on vancomycin and ceftriaxone. Vancomycin was discontinued after 48 hours, and ceftriaxone was changed to cefepime. Cultures on HD 13 grew budding yeast, and antifungal therapy with fluconazole was initiated. A new PICC line and hemodialysis catheter were subsequently placed. On HD 17, cultures grew gram-negative rods, prompting the addition of ciprofloxacin. Capsofungin was initiated following the identification of gram-negative rods and persistent fungemia. He was prophylactically started on a regimen of linezolid, meropenem, and pentamidine. Despite escalation of antifungals to voriconazole and repeated removal and replacement of the PICC line and hemodialysis catheter, blood cultures continued to demonstrate budding yeast. Ongoing candidemia prompted transition to amphotericin B on HD 35. Despite maximal antifungal therapy and line management, fungemia persisted, contributing to progressive multiorgan failure.

**Figure 4:**
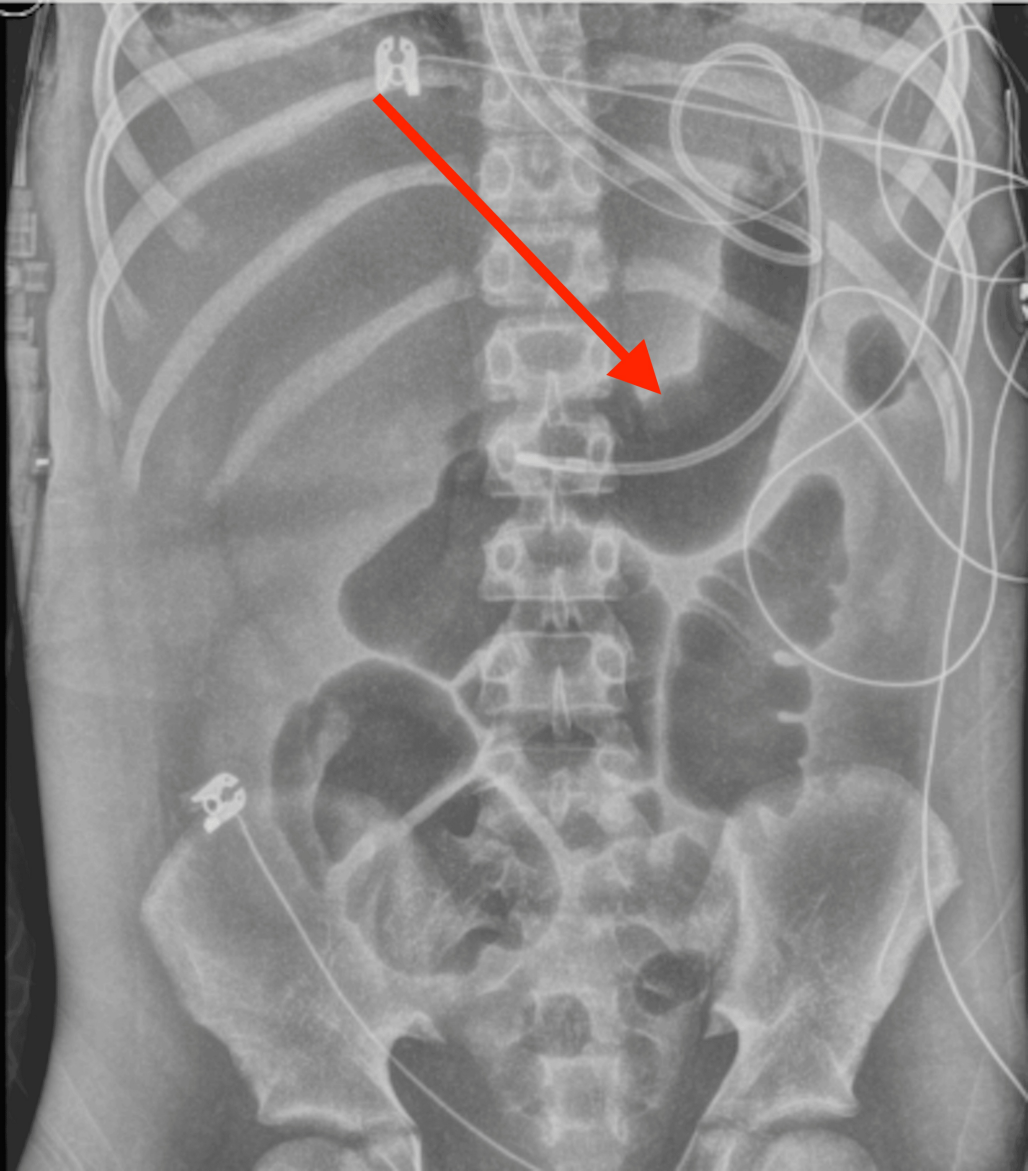
Portable supine abdominal radiograph suggesting ileus This image shows multiple dilated gas-filled bowel loops suggestive of ileus in the appropriate clinical context. An enteric tube projects over the stomach.

His course was complicated by moderate circumferential pericardial effusion without evidence of tamponade, bilateral pleural effusions, ascites, hepatic dysfunction, and progressive coagulopathy, consistent with evolving multiorgan failure (Figure 4).

Despite aggressive multidisciplinary management involving pediatric oncology, critical care, nephrology, infectious disease, cardiology, and gastroenterology teams, the patient’s condition continued to deteriorate. He ultimately died from refractory fungal sepsis with multiorgan failure on HD 46.

## Discussion

According to Burkhardt and Hermison, up to 60% of reported T-ALL cases present with bulky mediastinal disease in pediatric patients, placing affected individuals at risk for airway compromise and rapid cardiopulmonary deterioration [11]. In this case, early symptoms were nonspecific and initially treated as pneumonia, delaying recognition of a large anterior mediastinal mass until the patient presented in extremis. Similar delays in diagnosis have been described in pediatric patients whose early manifestations of lymphoma or leukemia mimic common respiratory infections [12].

Airway compromise associated with anterior mediastinal masses represents a well-recognized anesthetic and critical care challenge. Supine positioning and sedation may precipitate sudden airway collapse or cardiovascular compromise due to extrinsic compression of the tracheobronchial tree or great vessels [10]. In the present case, emergent airway intervention was unavoidable given the severity of hypoxemia and altered responsiveness, highlighting the importance of early identification and multidisciplinary planning when mediastinal pathology is suspected.

Management of the airway in patients with anterior mediastinal masses is challenging due to the risk of dynamic airway and cardiovascular compromise. Reductions in airway tone and changes in positioning can exacerbate compression of the central airway and surrounding vascular structures. In this case, the severity of hypoxemia and altered mental status necessitated urgent intervention despite these risks, highlighting the difficulty of balancing physiologic stability with the need for immediate airway control.

The patient’s course was further complicated by severe TLS, a life-threatening oncologic emergency resulting from rapid tumor cell breakdown following initiation of cytotoxic therapy. TLS is characterized by hyperuricemia, hyperphosphatemia, hypocalcemia, and acute kidney injury and is more likely in patients with high tumor burden, hyperleukocytosis, or steroid-sensitive malignancies such as T-ALL [13]. Although TLS is often preventable with early recognition and prophylactic measures, severe cases may still progress to oligo-anuric renal failure requiring renal replacement therapy, as occurred in this patient [13]. These metabolic derangements reflect rapid cellular breakdown rather than a primary endocrine disturbance, although they can secondarily disrupt multiple organ systems, including renal and cardiopulmonary function.

In addition to metabolic complications, induction chemotherapy placed the patient at significant risk for infectious morbidity. Profound neutropenia during induction is a known risk factor for severe infections in pediatric leukemia patients [14]. *Candida* species remain an important cause of invasive fungal infection in immunocompromised children and are associated with high mortality despite antifungal therapy, particularly when infections are persistent or line-associated [14]. In this case, refractory *Candida albicans* fungemia was the primary driver of progressive multiorgan failure and death.

The patient also developed acute lung injury with features consistent with acute respiratory distress syndrome, likely reflecting a multifactorial process involving capillary leak, fluid overload related to renal failure, systemic inflammation, and sepsis [15]. Additional complications, including neutropenic enterocolitis, venous thrombosis, serosal effusions, and hepatic dysfunction, further illustrate the complexity of managing overlapping oncologic and critical care emergencies.

This case underscores the necessity of close collaboration among pediatric emergency medicine, oncology, critical care, nephrology, infectious disease, cardiology, and gastroenterology teams. Multidisciplinary care models have been shown to improve coordination, safety, and outcomes in children with complex oncologic presentations requiring intensive care support [16]. Early recognition of mediastinal disease, prompt initiation of supportive care, and proactive management of treatment-related complications remain essential to improving outcomes in high-risk pediatric leukemia patients.

This case illustrates the dangerous intersection of delayed oncologic diagnosis, high tumor burden, and induction-related complications in T-ALL. While mediastinal masses are well recognized in T-ALL, the subtlety of early symptoms may create a false sense of clinical stability. In patients with progressive fatigue, weight loss, and persistent respiratory complaints unresponsive to antibiotics, earlier chest imaging may have altered the diagnostic timeline. Furthermore, this case emphasizes how initial stabilization does not eliminate risk; rather, it transitions patients into a second high-risk phase characterized by tumor lysis, immunosuppression, and infection. The cascade from mediastinal compression to TLS, acute kidney injury, neutropenia, invasive fungal infection, and multiorgan failure reflects the compounding nature of pediatric oncologic emergencies. Earlier recognition and imaging in patients with persistent or atypical respiratory symptoms may alter the diagnostic trajectory and potentially reduce the risk of life-threatening complications.

This report describes a single patient, which limits the generalizability of findings regarding diagnostic timing, management decisions, and outcomes in pediatric T-ALL. As a retrospective case review, the analysis relies on documentation within the electronic medical record, and certain laboratory values and detailed physiologic parameters were not consistently available for comprehensive longitudinal assessment.

The complexity of the patient’s course, characterized by overlapping oncologic, metabolic, infectious, and critical care complications, precludes determining the relative contribution of any single factor to the fatal outcome. Additionally, management occurred within a tertiary pediatric intensive care setting with access to advanced multidisciplinary resources, which may not reflect practice in other environments.

As with all case reports, causal inferences cannot be established; this report highlights a high-risk clinical scenario rather than implying that such outcomes are typical in pediatric T-ALL.

## Conclusions

This case highlights the importance of maintaining a high index of suspicion for mediastinal leukemia in pediatric patients presenting with persistent respiratory or systemic symptoms unresponsive to standard antibiotic therapy. Early chest imaging and prompt hematologic evaluation are critical to reducing the risk of sudden airway compromise and life-threatening metabolic complications. It further demonstrates the potential for rapid multisystem deterioration in T-ALL, even in previously healthy children, and underscores the importance of early recognition, aggressive stabilization, and coordinated multidisciplinary management in pediatric oncologic emergencies. While this represents a single case, it reinforces key clinical considerations in recognizing and managing high-risk pediatric patients with suspected mediastinal malignancy.
